# Health belief model and cervical cancer screening intention among lower socioeconomic women in Malaysia: a pilot study

**DOI:** 10.3389/fpubh.2026.1843558

**Published:** 2026-06-12

**Authors:** Siti Nur Farhana Harun, Arina Anis Azlan, Nor Zam Azihan Mohd Hassan, Emma Mirza Wati Mohamad

**Affiliations:** 1 Centre for Research in Media and Communication, Faculty of Social Sciences and Humanities, Universiti Kebangsaan Malaysia, Bangi, Malaysia; 2 Komunikasi Kesihatan (Healthcomm) – UKM Research Group, Universiti Kebangsaan Malaysia, Bangi, Malaysia; 3 Institute for Health Behavioural Research, National Institutes of Health, Ministry of Health Malaysia, Setia Alam, Selangor, Malaysia; 4 Institute for Health System Research, National Institutes of Health, Ministry of Health Malaysia, Setia Alam, Selangor, Malaysia

**Keywords:** cervical cancer, health belief model, lower socioeconomic group, preventive health behavior, screening intention

## Abstract

**Background:**

Cervical cancer remains a major public health concern in Malaysia, particularly among women from lower socioeconomic groups. Despite the availability of free Pap smear screening, uptake remains low. Understanding psychological factors influencing screening intention is important for informing contextually appropriate interventions. This pilot study explored the relevance of Health Belief Model (HBM) constructs in relation to screening intention while assessing the validity and reliability of the adapted instrument.

**Methods:**

A cross-sectional pilot study was conducted between April to May 2025 involving 54 women aged 20 to 65 years from the lower socioeconomic status women residing in the Klang Valley, Malaysia. Participants completed a self-administered questionnaire comprising sociodemographic information and six constructs of the HBM. Screening intention was measured using a 11-point Likert scale. Data were analyzed using descriptive statistics, bivariate analysis and multivariate logistic regression.

**Results:**

No statistically significant association between screening intention and any sociodemographic variables. However, significant differences in Health Belief Model constructs were identified across selected sociodemographic characteristics. Multivariate logistic regression showed that perceived barriers (OR = 0.848, *p* = 0.020) and perceived severity (OR = 0.782, *p* = 0.044) were significantly associated with screening intention in the multivariate model.

**Conclusion:**

This pilot study examined the preliminary relevance of Health Belief Model constructs and evaluated the validity and reliability of the adapted instrument. The findings suggest that perceived barriers and perceived severity may influence screening intention; however, given the small and exploratory sample, the results are not generalizable. In addition, these findings provide a basis for refining the instrument and informing a larger, adequately powered study.

## Introduction

Cervical cancer is one of the most preventable forms of cancer, yet it remains a major public health concern (1). In 2022, approximately 662,044 cases of cervical cancer were reported globally, with an age-standardized incidence rate of 14.12 per 100,000, and about 348,709 deaths, corresponding to an age-standardized mortality rate of 7.08 per 100,000. Cervical cancer ranks as the fourth leading cause of cancer-related morbidity and mortality among women worldwide (2). In Malaysia, cervical cancer ranks as one of the most common cancer overall, accounting for 4.2% of all cancer cases. The pap smear is one of the screening methods and provided free of charge at primary healthcare facilities. It is recommended for all women aged 20 to 65 years who are currently or have previously been sexually active (3, 4). Despite wide accessibility, national screening coverage remains inadequate (3) particularly among women from lower socioeconomic backgrounds (5). The National Health and Morbidity Survey (NHMS) 2023 reported the lowest screening uptake among this group (6). Barriers include limited awareness of screening services, fear of the procedure or diagnosis, fear of positive results (7) and lack of social support (8). In addition to these structural and emotional factors, behavioral intention plays a crucial role in determining whether individuals take action on health recommendations. Studies have shown that behavioral intention is one of the most significant predictors of actual health behavior including participation in screening programs (9). Importantly, individuals’ health beliefs can influence their health intentions and subsequent behaviors.

**Figure 1:**
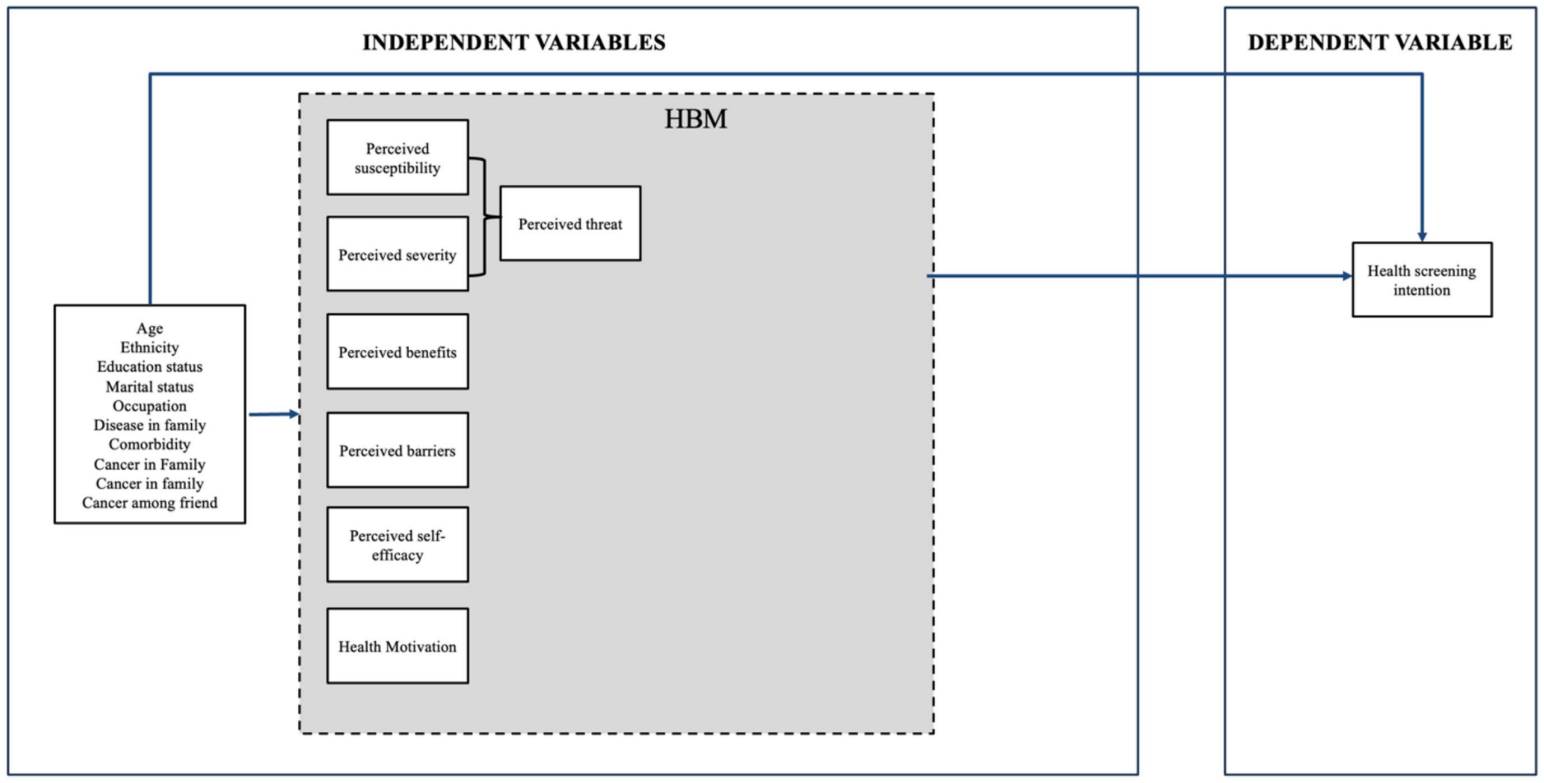
Explanatory variables associated with screening intention. Components of the health belief model. Glanze et al. (82).

One of the most widely used frameworks for understanding these beliefs is the Health Belief Model (HBM), developed in the 1950s by Hochbaum, Rosenstock, and Kegels (10), extensively used to explain preventive health behavior (11). It suggests that individuals are more likely to take preventive action when they perceive themselves at risk, recognize benefits and feel motivated to act (12). The HBM has been applied across various health behaviors, including screening and immunization uptake (10, 13). The model includes six key constructs, namely perceived susceptibility, perceived severity, perceived benefits, perceived barriers, cues to action and self-efficacy (Figure 1). These constructs describe how individuals evaluate health threats and the factors that support or hinder health action (14, 15). This study also incorporates health motivations, which was introduced by Becker, as supplementary construct reflecting general motivation to maintain or improve health (16). When used alongside the original HBM constructs, health motivation has demonstrated significant predictive value in understanding preventive health behaviors (16, 17).

Health belief model constructs have been widely validated in cervical cancer screening studies. Perceived barriers consistently emerge as a strong negative predictor of screening uptake (18–22). Perceived susceptibility and perceived benefits have also been shown to influence the likelihood of screening participation (23–25) while self-efficacy (25) and perceived severity (22) have also demonstrated predictive value, indicating confidence in one’s ability to undergo screening.

Empirical studies provide further support for these relationships. For instance, AlShamlan, AlOmar (18) reported that higher perceived barriers significantly reduced the likelihood of screening uptake among female healthcare workers in Saudi Arabia, underscoring the dominant role of psychological and structural constraints even among medically informed populations. Similarly, Ampofo, Adumatta (19) demonstrated that multiple HBM constructs, including perceived benefits, perceived threat, cues to action and perceived barriers, collectively influence screening participation. Their findings further revealed that negative beliefs, attitudes and socioeconomic as well as healthcare system barriers remain critical obstacles to screening uptake. Importantly, evidence from a systematic review Lau, Lim (26) indicates that HBM constructs are consistently associated with screening participation and can effectively predict preventive health behavior. Taken together, these findings highlight the practical relevance of the HBM as a comprehensive framework for understanding and predicting cervical cancer screening uptake, supporting its use in guiding both research and intervention strategies.

Although the HBM has been widely used to examine screening behaviors and its constructs have been consistently associated with screening intention, existing studies show considerable variability in how these constructs are measured and applied and limited attention has been given to how sociodemographic differences shape variations in health beliefs across population subgroups. In particular, evidence remains insufficient regarding how these variations manifest among lower socioeconomic populations, who are often underrepresented in screening research.

This study was conducted in Klang Valley, a major metropolitan region in central Peninsular Malaysia encompassing Kuala Lumpur and surrounding districts. The area forms a densely populated urban region with notable socioeconomic disparities (27). This persistent gap in uptake highlights the need to better understand the underlying factors influencing screening decisions, particularly the role of health beliefs and intentions. Therefore, it is essential to explore the relationship between HBM constructs and cervical cancer screening intention among this vulnerable group. Hence, this pilot study aims to examine the relevance of HBM constructs to cervical cancer screening intention among Malaysian women from lower socioeconomic backgrounds. The study also seeks to evaluate the preliminary validity and reliability of the instrument to inform a larger-scale future study.

Building on this, behavioral models such as the HBM have been widely recognized for their ability to identify the sociocognitive factors influencing women’s screening decisions and to guide the development of targeted interventions (28). Findings from this pilot study therefore will inform the subsequent phase of the larger study, which aims to develop and evaluate a theory-driven health promotional intervention to improve cervical cancer screening uptake among women in the target population. Specifically, the insights generated will help identify key beliefs and perceptions related to screening and inform how these can be systematically addressed using the HBM as the guiding theoretical framework (refer to the Supplementary material for details on the research phases).

## Materials and methods

### Study design and setting

A cross-sectional study design was carried out as a pilot study between 1st April to 5th May 2025 in the Klang Valley, Malaysia.

### Participants and sampling

Participants were recruited using convenience sampling. Eligibility criteria included Malaysian woman aged 20 to 65 years, residing in the Klang Valley and belong to the lower socioeconomic group, defined as having a household income below RM5,250 per month in accordance with the Malaysian B40 classification (29). In Malaysia, household income is typically divided into three categories: the Bottom 40 percent (B40) with a monthly income of RM5,249 (about $1,293 USD) or less; the Middle 40 percent (M40), earning between RM5,250 (roughly $1,294 USD) and RM11,819 (around $2,912 USD); and the Top 20 percent (T20) with monthly income exceeding RM11,819 (29). Respondents also included individuals who had either never undergone a Pap smear or had not complied with Ministry of Health Malaysia screening recommendations for more than *3* years. Pregnant women were excluded as they are generally not advised to undergo cervical cancer screening during this period. Women with a history of cervical cancer, previous hysterectomy or known gynecological malignancies were also excluded. In addition, individuals who were medically unstable, unable to provide informed consent, or had significant cognitive or communication difficulties that prevented questionnaire completion were not eligible for the study. Participants were recruited from community settings across the Klang Valley, reflecting the urban focus of the study population. Eligible B40 women were approached based on availability and willingness to participate. Written informed consent was obtained after the study objectives were explained. Participants were then given a self-administered questionnaire to complete, with the researcher present to clarify any questions if needed.

A total of 54 respondents participated in the study, in line with recommended guidelines for pilot testing, which suggest a sample size between 30 and 500 participants (30, 31). Consistent with established methodological recommendations, pilot studies typically require between 10 and 40 participants, which is sufficient to provide estimates precise enough for a range of different objectives (32). To accommodate an anticipated attrition rate of approximately 20%, the target was adjusted to 50 participants. Ultimately, 54 participants were recruited, which exceeds the recommended minimum for pilot study.

### Instrument

This questionnaire was adopted from the Malay version of the Champion’s Health Belief Model Scale (CHBMS) for breast cancer, previously validated by Parsa, Kandiah (33) and Htay, Schliemann (34). The validated CHBMS by Chisale Mabotja et al. (22) was also used as a reference. Permission to use and validate the CHBMS was obtained from the original author of Champion’s Health Belief Model Scale (16). The instrument demonstrated good internal consistency with a Cronbach’s alpha of 0.821. The questionnaire has three sections, with the first section being the demography section, followed by the Health Belief Model Scale for Cervical Cancer and Pap Smear Screening, which has six subsections. The final section assesses the intention to undergo cervical cancer screening. This intention was measured by asking participants a single question: “What is your likelihood of undergoing cervical cancer screening?” Responses were recorded on a 11-point Likert scale, ranging from 0 (no intention) to 10 (strongest intention). Participants’ intentions were then classified as “low” or “high” based on the median score, which served as the cut-off point. This approach of using a median score to categorize health behavior intentions has been employed in previous studies (35, 36).

### Content validity assessment

Content validity measures the extent to which the instrument appropriately captures the intended subject matter (37). It ensures that the questionnaire items are relevant, comprehensive and appropriate for the construct of interest. In this study, content validity was evaluated through expert review using the Content Validity Index (CVI) to support the validity of an assessment tool. To establish content validity, the drafted questionnaire was reviewed by the six subject matter experts in accordance with recommendations suggesting a minimum of six reviewers for content validation (38). The expert panel comprised three public health specialists, one Obstetrics and Gynaecology consultant and two family medicine specialists.

The evaluation focused on assessing the relevance and clarity of the instrument’s content to ensure its alignment with the cervical cancer screening guidelines established by the Ministry of Health Malaysia. Subject matter experts (SMEs) were provided with a structured evaluation checklist for each item, using the Content Validity Index (CVI) form. The CVI involves individual expert rating each item on a 4-point scale, ranging from 1 (not relevant) to 4 (highly relevant). The CVI for each item was then calculated as the proportion of experts who rated the item as either 3 or 4, indicating acceptable relevance. Items that received an the score of 0.83 or higher were considered to have an acceptable CVI value, in line with the recommendation by Lynn (39). To assess content validity, the Content Validity Index (CVI) was calculated using formulas for I-CVI, S-CVI/Ave, and S-CVI/UA as illustrated by Yusoff (40).

The I-CVI for the CHBM was 0.97 which is above the acceptable value of 0.83 (40, 41) (refer Supplementary material).

### Face validity assessment

Face validity assesses whether the instrument appears appropriate, relevant, and meaningful from the perspective of the target population. It ensures that the questionnaire items are perceived as clear, comprehensible and contextually appropriate (42). Clarity relates to whether the instructions and wording of the items can be interpreted in intended way, without confusion or multiple meanings. Comprehensibility, on the other hand, refers to the ease with which raters can understand the language and sentence construction. Establishing response process validity is a key step in confirming the overall validity of instruments like questionnaires, especially in research settings. This form of validity can be measured using the Face Validity Index (FVI), which has been applied in various studies to support the validation of assessment tools (41).

A total of 10 respondents aged 20 to 65 years from the lower socioeconomic status (B40) women, selected to reflect the characteristics of the actual study participants to evaluate the clarity and comprehensibility of each item in the questionnaire. Each participant independently assessed the items using a 4-point Likert scale: 1 = not clear, 2 = somewhat clear, 3 = clear, and 4 = very clear. According to Mohamad Marzuki, Yaacob (43), a minimum cut-off score of 0.83 is required when using 10 raters to indicate acceptable face validity. To assess face validity, the Face Validity Index (FVI) was calculated using formula for The Item-level Face Validity Index (I-FVI), S-FVI/Ave (scale-level face validity index based on the average method), and S-FVI/UA (scale-level face validity index based on the universal agreement method) as illustrated by Yusoff (41).

The I-FVI for the CHBM was 0.96 which is above the acceptable value of 0.83 (40, 41) (refer Supplementary material).

### Reliability testing

Reliability can be assessed by examining internal consistency to determine whether scale items contribute to the measured construct. Internal consistency is estimated using indices such as coefficient alpha (Cronbach’s alpha) (44, 45) as cited in Larsson, Engström (46).

Cronbach’s alpha is commonly used to assess the internal consistency of a scale. The coefficient indicates how strongly the items are related to one another, showing whether they measure a single, consistent construct (47, 48) as cited in (49). Nunnally (50) proposed minimum reliability standards of 0.80 for basic research and 0.90 for applied research. However, it is common among modern researchers to consider reliabilities within the 0.60 and 0.70 as acceptable or satisfactory (51) as cited in (44). The Cronbach’s alpha value for the instrument is presented in Table 1.

**Table 1:** Cronbach’s alpha values.

HBM constructs	Number of items	Cronbach’s alpha pilot test (*n* = 54)
1. Total	35	0.821
2. Benefit	6	0.898
3. Barriers	7	0.863
4. Severity	4	0.847
5. Susceptibility	4	0.899
6. Health Motivation	4	0.918
7. Self-Efficacy	10	0.942

### Data collection

Data were collected through guided self-administered questionnaires distributed to eligible participants. Data were collected using a guided self-administered approach, in which trained researcher provided standardized guidance during questionnaire completion. This approach minimized comprehension barriers and ensured accurate administration among respondents with lower educational background and older adults.

Prior to participation, written informed consent was obtained from all respondents. To ensure data quality, a validated data collection tool was used alongside a guided self-administered approach, helping respondents clearly understand each item. Trained researchers were present during data collection to clarify any questions and ensure consistent administration. Upon completion, all questionnaires were carefully checked for completeness and accuracy before data entry and analysis.

### Variables descriptions

#### Dependent variable

The dependent variable, screening intention, was measured using a 11-point Likert scale (0 = lowest, 10 = highest). For analysis, responses were categorized into low intention (<7) and high intention (≥7), using the median score as the cutoff point. This approach was adopted to facilitate a simplified and interpretable analysis consistent with the exploratory nature of this pilot study. The binary specification facilitated clearer interpretation of results in terms of likelihood of higher versus lower intention, which is useful for identifying potential determinants to inform subsequent intervention development. This approach is consistent with prior study that have operationalized behavioral intention as a dichotomous outcome (35).

#### Explanatory variables

Several explanatory variables were included in the analysis, as illustrated in Figure 1. Sociodemographic variables comprised of ethnicity (0 = Malay, 1 = Chinese, 2 = Indian), marital status (0 = not married, 1 = married), educational level (0 = primary, 1 = secondary, 2 = tertiary), and occupational sector [0 = government, 1 = private (including private sector, self-employed and informal employment), 2 = others (including housewife and unemployed)]. Age was categorized into four groups: 0 = ≤35 years, 1 = 36 to 45 years, 2 = 46 to 55 years and 3 = more than 55 years. Additional binary variables reflecting personal and familial health history (self-reported) were also included, namely disease in family (0 = No, 1 = Yes), comorbid (0 = No, 1 = Yes), cervical cancer in family (0 = No, 1 = Yes), other cancer in family (0 = No, 1 = Yes) and cervical cancer among friends (0 = No, 1 = Yes). The inclusion of history of other cancer in the family as explanatory variables was because family cancer experience can influence an individual’s perception of personal risk and perceived susceptibility toward cervical cancer. The inclusion of cervical cancer among friends as an explanatory variable was to capture social exposure to the disease which may influence individuals’ perceptions of risk and screening-related behavior through peer interaction and shared experiences. Evidence suggests that individuals with family members diagnosed with cancer often demonstrate heightened risk awareness, greater health-seeking behavior, and increased motivation for preventive screening. Therefore, this variable was added to capture potential predictor in screening intention that may arise from prior family exposure to cancer (52).

In addition, six constructs from the HBM were included as continuous variables. The six constructs are perceived barriers, perceived benefits, perceived motivation, perceived severity, self-efficacy and perceived susceptibility. These were measured using a validated self-administered questionnaire with items rated on a 5-point Likert scale (1 = strongly disagree to 5 = strongly agree). Construct scores were computed as the sum of items within each domain.

### Statistical analysis

The data were analyzed using Stata software version 18 (Stata Corp, College Station, Texas, USA) and SPSS version 29. Initial descriptive statistics were performed to explain the characteristics of the respondents. The determining factors of screening intention were identified using bivariate and multivariate statistical analysis. Bivariate analyses were performed to assess the associations between screening intention and the HBM constructs across different sociodemographic status and screening intention. Subsequently, a logistic regression model was employed to identify determinants of screening intention.

Logistic regression is the preferred analytical method when the dependent variable is binary or dichotomous in nature (53). In this analysis, screening intention was treated as a binary outcome variable, coded as 0 for low intention and 1 for high intention. Variables with a *p*-value < 0.25 in the bivariate analysis were selected as explanatory variables for inclusion in the multivariable logit regression model (54).

The mathematical model of the study is expressed as the following Equation 1:


$$\text{Logit}(Y_{i})=\log O_{i}=\log(\frac{p_{i}}{1-p_{i}})=\beta_{0}+\beta_{1}X_{i1}+\ldots +\beta_{p}X_{\mathrm{ip}}+u_{i}$$ (1)

Where *p*_i_ is the probability of having screening intention. X_ip_ refers to explanatory variables included in the model. $u_{i}$ is the random error term, and *β* is a beta coefficient to be estimated. In this model, the regression coefficients (β) were exponentiated as expressed as odds ratios (e^βp^), facilitating interpretation of the effect size. The adjusted logistic regression equation in terms of odds ratios is therefore expressed as the Equation 2:


$$Y_{i}=O_{i}=\frac{p_{i}}{1-p_{i}}=e^{\beta_{0}}+e^{\beta_{1}}X_{i1}+\ldots +e^{\beta_{p}}X_{\mathrm{ip}}+u_{i}$$ (2)

The significance level was set at 0.05, and all *p*-values were based on two-tailed tests.

### Ethical approval

This research has been registered under the National Medical Research Registration [NMRR ID-24-03835-XDC], with ethics approval from the Medical Research Ethics Committee (MREC) on 21 February 2025 (Ref: 24-03835-XDC) and the Research Ethics Committee of Universiti Kebangsaan Malaysia [JEP-2024-1123]. Written informed consent was obtained from all participants prior to their participation in the study.

## Results

### Sociodemographic and screening intention

Table 2 presents the descriptive characteristics and bivariate analyses of screening intention across sociodemographic variables. Most respondents were Malay, married, and had tertiary education, with employment distributed mainly between the private and government sectors. Bivariate analysis using Chi-square or Fisher’s exact tests showed no statistically significant associations between screening intention and any sociodemographic variables, including ethnicity, age group, marital status, education level, occupational sector, presence of comorbidities, family history of disease or cancer, or friends with cervical cancer (all *p* > 0.05).

**Table 2:** Sociodemographic and screening intention (*n* = 54).

Sociodemographic	*N* (%)	Low intention		High intention		*p*-value (two tailed)
		*n*	%	*n*	%	
Ethnicity^$^						0.250
Malay	40 (74.1)	17	42.5	23	57.5	
Chinese	5 (9.3)	1	20.0	4	80.0	
Indian	9 (16.6)	6	66.7	3	33.3	
Age group^$^						0.880
35 years and less	22 (40.7)	11	50.0	11	50.0	
36–45 years	21 (38.9)	8	38.1	13	61.9	
46–55 years	6 (11.1)	3	50.0	3	50.0	
More than 55 years	5 (9.3)	2	40.0	3	60.0	
Marital status^¶^						0.434
Not married	13 (24.1)	7	53.9	6	46.2	
Married	41 (75.9)	17	41.5	24	58.5	
Education level^$^						0.585
Primary	3 (5.6)	2	66.7	1	33.3	
Secondary	16 (29.6)	8	50.0	8	50.0	
Tertiary	35 (64.8)	14	40.0	21	60.0	
Occupational sector^$^						0.648
Government	22 (40.7)	11	50.0	11	50.0	
Private	25 (46.3)	11	44.0	14	56.0	
Others	7 (13.0)	2	28.6	5	71.4	
Disease in family^¶^						0.851
No	33 (61.1)	15	45.5	18	54.6	
Yes	21 (38.9)	9	42.9	12	57.1	
Comorbid^¶^						0.454
No	33 (61.1)	16	48.5	17	51.5	
Yes	21 (38.9)	8	38.1	13	61.9	
Cervical cancer in family^$^						0.682
No	48 (88.9)	22	45.8	26	54.2	
Yes	6 (11.1)	2	33.3	4	66.7	
Other cancer in family^¶^						0.165
No	40 (74.1)	20	50.0	20	50.0	
Yes	14 (25.9)	4	28.6	10	71.4	
Cervical cancer among friends^$^						0.639
No	45 (83.3)	20	44.4	25	55.6	
Yes	9 (16.7)	4	44.4	5	55.6	

^$^Fisher exact test. ^¶^Chi square test.

### Sociodemographic and health belief model constructs

Table 3 presents the six HBM constructs scores across different sociodemographic status. A statistically significant difference in perceived benefit scores was observed across age groups (<35 years: *n* = 11; 36–45 years: *n* = 8; 46–55 years: *n* = 3; more than 55 years: *n* = 2), χ^2^ (13, *N* = 54) = 51.814, *p* = 0.001. Older age groups 45–55 years (Median = 27.0) and more than 55 years (Median = 26.0) reported higher perceived benefit scores compared to younger respondents aged <35 years (Median = 24.0) and 36–45 years (Median = 24.0).

**Table 3:** Sociodemographic and health belief model constructs (*n* = 54).

Sociodemographic status		Perceived barrier	Perceived benefit	Health motivation	Perceived self-efficacy	Perceived severity	Perceived Susceptibility
Ethnicity							
Malay	Mean (SD)	16.76 (5.45)	–	–	–	13.38 (3.30)	–
	Median (IQR)	–	24.5 (6.0)	16.0 (3.0)	40.0 (6.5)	–	12.0 (1.0)
Chinese	Mean (SD)	14.60 (5.13)	–	–	–	12.00 (3.74)	–
	Median (IQR)	–	23.0 (2.0)	16.0 (3.0)	40.0 (5.0)	–	12.0 (4.0)
Indian	Mean (SD)	17.22 (8.11)	–	–	–	15.00 (4.82)	–
	Median (IQR)	–	24.0 (2.0)	16.0 (4.0)	39.0 (9.0)	–	12.0 (4.0)
*p*-value (two tailed)		^€^ 0.7111	^₦^0.7474	^₦^0.4863	^₦^0.8438	^€^0.3017	^₦^0.5232
Age group							
35 years and less	Mean (SD)	16.68 (6.24)	–	–	–	14.27 (3.41)	–
	Median (IQR)	–	24.0 (7.0)	16.0 (1.0)	40.0 (1.0)	–	12.0 (2.0)
36–45 years	Mean (SD)	16.52 (5.79)	–	–	–	13.14 (3.73)	–
	Median (IQR)	–	24.0 (8.0)	16.0 (4.0)	40.0 (8.0)	–	12.0 (3.0)
46–55 years	Mean (SD)	17.50 (7.74)	–	–	–	11.83 (4.92)	–
	Median (IQR)	–	27.0 (6.0)	17.5 (3.0)	39.5 (18.0)	–	12.0 (1.0)
More than 55 years	Mean (SD)	15.60 (2.07)	–	–	–	13.8 (2.39)	–
	Median (IQR)	–	26.0 (6.0)	16.0 (3.0)	33.0 (12.0)	–	12.0 (0.0)
*p*-value (two tailed)		^€^ 0.9633	^**₦**^ **0.0001** ^***^	^₦^0.3510	^₦^0.6368	^€^0.3017	^₦^0. 085
Marital status							
Not married	Mean (SD)	16.23 (7.24)	–	–	–	13.54 (4.98)	–
	Median (IQR)	–	24.0 (2.0)	16.0 (3.0)	40.0 (9.0)	–	11.0 (5.0)
Married	Mean (SD)	16.73 (5.44)	–	–	–	13.51 (3.17)	–
	Median (IQR)	–	24.0 (7.0)	16.0 (3.0)	40.0 (6.0)	–	12 (0.0)
*p*-value (two tailed)		^£^0.7908	^₩^0.6600	^₩^0.8760	^₩^0.7981	^₮^0.9859	^₩^ **0.0384** ^*^
Education level							
Primary	Mean (SD)	23.67 (1.53)	–	–	–	18.33 (2.89)	–
	Median (IQR)	–	24.0 (12.0)	16.0 (4.0)	39.0 (9.0)	–	12.0 (5.0)
Secondary	Mean (SD)	18.06 (6.09)	–	–	–	12.75 (3.59)	–
	Median (IQR)	–	24.0 (5.0)	16.0 (3.0)	40.0 (6.5)	–	12.0 (1.5)
Tertiary	Mean (SD)	15.34 (5.46)	–	–	–	13.46 (3.48)	–
	Median (IQR)	–	24.0 (6.0)	16.0 (4.0)	40.0 (8.0)	–	12.0 (3.0)
*p*-value (two tailed)		^€^ **0.0271** ^*^	^₦^0.9778	^₦^0.2434	^₦^0.9670	^€^ **0.0468** ^*^	^₦^0.9162
Occupation sector							
Government	Mean (SD)	16.23 (5.31)	–	–	–	13.86 (3.24)	–
	Median (IQR)	–	25.0 (6.0)	16.0 (4.0)	40.0 (7.0)	–	12.0 (3.0)
Private	Mean (SD)	16.44 (6.46)	–	–	–	13.48 (4.19)	–
	Median (IQR)	–	24.0 (7.0)	16.0 (2.0)	40.0 (5.0)	–	12.0 (1.0)
Others	Mean (SD)	18.43 (5.68)	–	–	–	12.57 (2.82)	–)
	Median (IQR)	–	25.0 (10.0)	16.0 (3.0)	34.0 (12.0)	–	12.0 (0.0)
*p*-value (two tailed)		^€^ 0.6814	^₦^0.9532	^₦^0.8208	^₦^0.0809	^€^ 0.7205	^₦^0.2675

^*^p < 0.05; ^**^p < 0.01; ^***^p < 0.001 (two tailed). ^€^One-Way ANOVA. ^₦^Kruskal Wallis Test. ^£^Independent *T*-Test equal variance. ^₮^Independent *T*-Test unequal variance. ^₩^Mann Whitney Test. Bold values indicate statistically significant results (*p* < 0.05). The symbol “–” in the table indicates that the corresponding value was not applicable or not reported because the data were presented using a different descriptive statistic format.

A statistically significant difference was observed in perceived susceptibility scores between married and not married respondents (z = −2.071, *p* = 0.0384), with the married group demonstrating a higher median score of perceived susceptibility (Median = 12.0) compared to the not married group (Median = 11.0).

A statistically significant difference was also observed in perceived barriers across education levels, *F*(2, 51) = 3.87, *p* = 0.0271. *Post-hoc* comparisons using the Tukey HSD test indicated that participants with primary education reported significantly higher perceived barriers score (Mean = 23.67, SD = 1.53) than those with tertiary education (Mean = 15.34, SD = 5.46).

Additionally, a significant difference in perceived severity scores was also found across education levels, *F*(2, 51) = 3.25, *p* = 0.0468. *Post-hoc* using Tukey’s HSD further revealed that individuals with primary education had significantly higher perceived severity scores (Mean = 18.33, SD = 2.89) compared to those with secondary (Mean = 12.75, SD = 3.59) and tertiary education (Mean = 13.46, SD = 3.48).

No statistically significant differences were observed in any of the HBM construct scores between ethnic categories and occupational sectors.

Table 4 presents a comparison of the six HBM constructs scores between health-related variables. A statistically significant difference was observed in perceived severity scores, whereby respondents without a family history of cervical cancer reported significantly higher perceived severity (Mean = 14.02, SD = 3.32) compared to those with a family history of cervical cancer (Mean = 9.50, SD = 3.78), *t*(52) = 3.10, *p* = 0.0031.

**Table 4:** Health-related variables and health belief model constructs (*n* = 54).

Health related variables		Perceived barrier	Perceived benefit	Health motivation	Perceived self-efficacy	Perceived severity	Perceived susceptibility
Disease in family							
No	Mean (SD)	17.21 (6.13)	–	–	–	13.67 (3.58)	–
	Median (IQR)	–	24.0 (5.0)	16.0 (3.0)	40.0 (3.0)	–	12.0 (3.0)
Yes	Mean (SD)	15.67 (5.40)	–	–	–	13.29 (3.80)	–
	Median (IQR)	–	26.0 (7.0)	16.0 (4.0)	40.0 (8.0)	–	12.0 (0.0)
*p*-value (two tailed)		^£^0.5773	^₩^0.2090	^₩^0.6481	^₩^0.8576	^£^0.7110	^₩^0.4397
Comorbid							
No	Mean (SD)	16.97 (6.35)	–	–	–	13.88 (2.98)	–
	Median (IQR)	–	24.0 (6.0)	16.0 (2.0)	40.0 (6.0)	–	12.0 (3.0)
Yes	Mean (SD)	16.05 (5.06)	–	–	–	12.95 (4.50)	–
	Median (IQR)	–	26.0 (6.0)	18.0 (4.0)	40.0 (9.0)	–	12.0 (1.0)
*p*-value (two tailed)		^£^0.3489	^₩^0.2508	^₩^0.4703	^₩^0.4040	^£^0.3659	^₩^0.6921
Cervical cancer in family							
No	Mean (SD)	17.13 (5.82)	–	–	–	14.02 (3.32)	–
	Median (IQR)	–	2.04 (6.0)	16.0 (3.0)	40.0 (7.5)	–	12.0 (1.5)
Yes	Mean (SD)	12.50 (4.68)	–	–	–	9.50 (3.78)	–
	Median (IQR)	–	24.5 (9.0)	17.0 (3.0)	40.0 (8.0)	–	11.5 (5.0)
*p*-value (two tailed)		^£^0.0675	^₩^0.9778	^₩^0.3052	^₩^0.9556	^£^ **0.0031** ^*^	^₩^0.4720
Other cancer in family							
No	Mean (SD)	17.05 (5.86)	–	–	–	14.15 (3.36)	–
	Median (IQR)	–	24.0 (6.0)	16.0 (3.0)	40.0 (6.5)	–	12.0 (1.5)
Yes	Mean (SD)	15.36 (5.85)	–	–	–	11.71 (3.91)	–
	Median (IQR)	–	26.5 (8.0)	16.0 (3.0)	40.0 (12.0)	–	12.0 (3.0)
*p*-value (two tailed)		^£^0.3564	^₩^0.3039	^₩^0.1520	^₩^0.8030	^£^ **0.0294** ^*^	^₩^0.9059
Cervical cancer among friends							
No	Mean (SD)	17.36 (5.76)	–	–	–	13.64 (3.53)	–
	Median (IQR)	–	24.0 (6.0)	16.0 (2.0)	40.0 (5.0)	–	12.0 (2.0)
Yes	Mean (SD)	12.89 (5.09)	–	–	–	12.89 (4.28)	–
	Median (IQR)	–	26.0 (9.0)	19.0 (4.0)	40.0 (20.0)	–	12.0 (1.0)
*p*-value (two tailed)		^£^0.0353	^₩^0.6220	^₩^0.1520	^₩^0.8695	^£^0.5739	^₩^0.9597

^*^p < 0.05; ^**^p < 0.01; ^***^p < 0.001 (two tailed). ^£^Independent *T*-Test equal variance. ^₩^ Mann–Whitney Test. Bold values indicate statistically significant results (*p* < 0.05).

A statistically significant difference was also found in perceived severity scores, with respondents without a family history of other cancer reporting significantly higher perceived severity (Mean = 14.15, SD = 3.36) compared to those with a family history of other cancer (Mean = 11.71, SD = 3.91), *t*(52) = 2.24, *p* = 0.0294.

No statistically significant differences were found in other HBM constructs scores based on the presence of comorbidities, a family history of disease, family history of other cancer, or friends diagnosed with cervical cancer.

### Health belief model and screening intention

Table 5 presents the comparison of HBM constructs scores between individuals with high and low screening intention, analyzed using independent t-tests or Mann–Whitney tests where appropriate. A statistically significant difference was observed in perceived barrier scores, with the low intention group reporting significantly higher barriers (Mean = 19.38, SD = 5.22) compared to the high intention group (Mean = 14.40, SD = 5.44), *t*(52) = 3.40, *p* = 0.0013. Similarly, the low intention group had significantly higher perceived severity scores (Mean = 15.08, SD = 3.30) than the high intention group (Mean = 12.27, SD = 3.44), *t*(52) = 3.04, *p* = 0.0037. There were no statistically significant differences in scores of perceived benefits, health motivation, self-efficacy, and perceived susceptibility between respondents with high and low screening intention.

**Table 5:** Health Belief Model scores and Screening intention (*n* = 54).

HBM	*n*	Low intention		High intention		*p-*value (two tailed)
		Mean (SD)	Median (IQR)	Mean (SD)	Median (IQR)	
Perceived barrier^Ψ^	54	19.38 (5.22)	-	14.40(5.44)	-	**0.0013** ^**^
Perceived benefit^₩^	54	-	24.0 (6.5)	-	25.5 (4.0)	0.2178
Health motivation^₩^	54	-	16.0 (2.0)	-	16.0 (3.0)	0.0748
Perceived self-efficacy^₩^	54	-	38.0 (7.5)	-	40.0 (3.0)	0.0517
Perceived severity^Ψ^	54	15.08(3.30)	-	12.27(3.44)	-	**0.0037** ^**^
Perceived susceptibility^₩^	54	-	12.0 (1.5)	-	12.0 (3.0)	0.8127

^*^p < 0.05; ^**^p < 0.01; ^***^p < 0.001. ^Ψ^Independent *T*-Test. ^₩^Mann-Whitney Test. Bold values indicate statistically significant results (*p* < 0.05).

### Determinants of screening intention

Table 6 shows the results of the Logit regression model of the screening intention determinants among the B40 population in Malaysia. The model includes six explanatory variables, selected based on bivariate analysis with a significance threshold of *p* < 0.25 (54). These variables were five variables from Champion’s Health Belief Model Scale constructs namely the perceived barriers, perceived benefits, perceived motivation, perceived severity and self-efficacy. Perceived Susceptibility was not included in the model as the *p*-value for bivariate analysis was more than 0.25. Another variable was history of other cancer in the family. Multicollinearity diagnostics were performed for all independent variables prior to conducting the logistic regression analysis. The pairwise Pearson correlation coefficients were all below 0.90, indicating the absence of excessively strong linear relationships. In addition, the Variance Inflation Factor (VIF) values for all predictors were consistently less than 5, further confirming that multicollinearity was not a concern in the model.

**Table 6:** Logit regression model of screening intention determinants among B40 population in Malaysia (*n* = 54).

Variables	*B* (Adj)	OR	SE	95% CI	*P-*value (two tailed)
Health belief model					
Perceived barrier	−0.165	0.848	0.071	(0.738, 0.974)	**0.020** ^*^
Perceived benefit	−0.116	0.891	0.105	(0.725, 1.093)	0.268
Health motivation	0.167	1.181	0.168	(0.850, 1.643)	0.321
Perceived self-efficacy	0.038	1.039	0.061	(0.921, 1.171)	0.534
Perceived severity	−0.246	0.782	0.122	(0.616, 0.992)	**0.044** ^*^
Cancer in family					
No (ref.)		–	–	–	–
Yes	0.395	1.494	1.243	(1.494, 1.243)	0.629
Constant	5.038	154.185	3.339		0.132

*p < 0.05, **p < 0.01, ***p < 0.001 (significance level at 5, 1, and 0.1% respectively). Cox-Snell, *R* squared = 0.310, Nagelkerke, *R* squared = 0.415. χ2 = 20.01, *p* = 0.0028. OR is Odds Ratio, CI is Confidence Interval, SE is Standard Error. Bold values indicate statistically significant results (*p* < 0.05).

Prior to model estimation, separation and coefficient stability were assessed using Stata 18 by examining cross-tabulations, convergence status and the magnitude of coefficients and standard errors. No complete or quasi-complete separation was observed. The model converged successfully and all parameter estimates were stable. Logistic regression analysis was therefore performed.

The model encompassing all the six explanatory variables was statistically significant, χ^2^ = 20.01, *p* = 0.0028. The model explained 31.0% (Cox-Snell R squared) to 41.5% (Nagelkerke R squared) of the variance in screening intention. The results showed that only two variables were statistically significant to determine screening intention, namely the perceived barriers and perceived severity. Respondents with higher perceived severity were 21.8% less likely to have screening intention (OR = 0.782). Additionally, respondents with higher perceived barriers were 15.2% less likely to have screening intention (OR = 0.848). Family history of other cancer, perceived benefits, health motivation and perceived self-efficacy were not statistically significant in determining screening intention.

### Item-level analysis of perceived barriers

An independent samples *t*-test was conducted to compare all seven perceived barrier item scores between women with low and high screening intention (Table 7) and the item-level analysis revealed that several specific barriers were significantly higher among women with low intention, indicating that these represent the most prominent hindrances within this population.

**Table 7:** Perceived barriers items scores between low and high intention.

Items for perceived barrier	Low intention Mean (SD)	High intention Mean (SD)	*t*	*P*-value (two tailed)	Effect size (eta squared)
(1) I would be ashamed to show my private parts to have a Pap smear test^£^	3.33 (1.129)	2.30 (1.179)	3.261	0.002^**^	0.1698
(2) Having a Pap smear test takes too much time^£^	2.75 (1.032)	2.2 (0.997)	1.984	0.053	0.0704
(3) I have other problems more important than having a Pap smear test in my life^₮^	2.92 (1.283)	2.13 (0.900)	2.535	0.015^*^	0.1100
(4) I am too old to have a Pap smear test regularly ^£^	2.08 (0.929)	1.73 (0.828)	1.463	0.150	0.0395
(5) I am afraid to undergo a Pap smear test because I do not understand what will be done ^£^	3.04 (1.233)	2.07 (1.172)	2.968	0.005^**^	0.1449
(6) I do not know how to get a Pap smear test ^£^	2.79 (1.215)	2.10 (1.094)	2.198	0.032^*^	0.0850
(7) I do not receive the support I need from close relatives to undergo a Pap smear test.^₮^	2.46 (1.179)	1.87 (0.86)	2.059	0.046^*^	0.0754

^*^p < 0.05; ^**^p < 0.01; ^***^p < 0.001 (two tailed). ^£^Independent T-Test equal variance. ^₮^Independent T-Test unequal variance.

Women with low intention reported significantly higher scores for Item 4 (*M* = 3.33, SD = 1.129) compared to those with high intention (*M* = 2.30, SD = 1.179), *t*(52) = 3.261, *p* = 0.002, with a large effect size (eta squared = 0.1698).

A similar pattern was observed for Item 16, where the low intention group (*M* = 3.04, SD = 1.233) scored significantly higher than the high intention group (*M* = 2.07, SD = 1.172), *t*(52) = 2.968, *p* = 0.005, also indicating a large effect size (eta squared = 0.1449).

For Item 10, women with low intention (*M* = 2.92, SD = 1.283) reported a significantly higher scores than those with high intention (*M* = 2.13, SD = 0.900), *t*(39.779) = 2.535, *p* = 0.015, with a moderate effect size (eta squared = 0.1100).

Similarly, Item 17 scores were significantly higher among women with low intention (*M* = 2.79, SD = 1.215) compared to the high intention group (*M* = 2.10, SD = 1.094), *t*(52) = 2.198, *p* = 0.032, with a moderate effect size (eta squared = 0.0850).

Item 18 also showed a significant difference, with higher scores in the low intention group (*M* = 2.46, SD = 1.179) compared to the high intention group (*M* = 1.87, SD = 0.86), *t*(40.892) = 2.059, *p* = 0.046, with a moderate effect size (eta squared = 0.0754).

All other perceived barrier items showed no significant difference between low and high intention.

### Item-level analysis of perceived severity

An independent samples t-test was conducted to compare all four perceived severity item scores between women with low and high screening intention (Table 8). The item-level analysis showed that several items were significantly higher among women with low screening intention compared to those with high intention, indicating that these represent the most prominent severity related perceptions within this population.

**Table 8:** Perceived severity items scores between low and high intention.

Items for perceived severity	Low intention Mean (SD)	High intention Mean (SD)	*t*	*P*-value (two tailed)	Effect size (eta squared)
(1) The thought of cervical cancer scares me^£^	4.13 (0.900)	3.23 (1.135)	3.138	0.003^**^	0.1592
(2) When I think about cervical cancer, my heart beats faster ^£^	3.71 (0.999)	2.90 (1.155)	2.711	0.009^**^	0.1238
(3) I am afraid to think about cervical cancer ^£^	3.83 (0.963)	3.07 (1.081)	2.717	0.009^**^	0.1243
(4) Problems I would experience with cervical cancer would last a long time^£^	3.42 (1.018)	3.07 (1.015)	1.258	0.214	0.0295

^*^p < 0.05; ^**^p < 0.01; ^***^p < 0.001 (two tailed). ^£^Independent *T*-Test equal variance.

Women with low intention reported significantly higher scores for Item 1 (*M* = 4.13, SD = 0.900) compared to those with high intention (*M* = 3.23, SD = 1.135), *t*(52) = 3.138, *p* = 0.003, with a large effect size (eta squared = 0.1592).

For Item 3, the low intention group (*M* = 3.83, SD = 0.963) scored significantly higher than the high intention group (*M* = 3.07, SD = 1.081), *t*(52) = 2.717, *p* = 0.009, indicating a moderate effect size (eta squared = 0.1238).

Similarly, Item 2 scores were significantly higher among women with low intention (*M* = 3.71, SD = 0.999) compared to the high intention group (*M* = 2.90, SD = 1.155), *t*(52) = 2.711, *p* = 0.009, with a moderate effect size (eta squared = 0.1238).

Item 4 did not show a statistically significant difference between the two groups.

## Discussion

### Sociodemographic and screening intention

This study aims to identify the relationship between sociodemographic factors, constructs of the HBM, and cervical cancer screening intention among lower socioeconomic Malaysian women. No statistically significant associations were found between screening intention and sociodemographic variables such as age, marital status, occupational sector, or education level. These findings are consistent with study conducted by Ebu (55), factors such as age, religion, marital status, and employment status were not significantly associated with screening intention. Studies have examined various sociodemographic factors as possible moderators of the relationship between intention and behavior, but most have found no significant effects. For example, Rhodes, Cox (56) reviewed various demographic factors such as age and gender and reported no consistent patterns across the physical activity studies they analyzed. This finding aligns with evidence suggesting that sociodemographic variables may not directly influence behavioral intention unless strengthen by cognitive and psychological constructs (57).

Although sociodemographic factors were not found to be significantly associated with screening intention in this study, the findings suggest a plausible indirect pathway. Sociodemographic characteristics may influence how individuals perceive susceptibility, severity, benefits, and barriers, as well as their level of motivation and confidence, which are key components of the HBM. These perceptual constructs, in turn, are more closely linked to screening intention (58). Therefore, the role of sociodemographic factors may operate indirectly through their influence on health beliefs rather than exerting a direct effect on intention.

In addition, the observed relationships between sociodemographic variables and HBM constructs suggest potential variation across population subgroups. Differences in age, educational level, marital status or health history may contribute to distinct patterns of health beliefs, which could subsequently influence health intention and behavior (59, 60). Even in the absence of a direct relationship, these subgroup variations remain important as they reflect how different segments of the population may differ in their perceptions and readiness to engage in screening. Collectively, these findings highlight the importance of considering indirect pathways and subgroup differences when interpreting the role of sociodemographic factors in preventive health behaviors.

### Sociodemographic and health belief model constructs

Bivariate analysis was conducted to examine whether sociodemographic factors such as age, marital status, education level, and health history were associated with the constructs of the HBM. This analysis aimed to explore whether individuals’ health beliefs, including perceived susceptibility, severity, benefits, barriers, health motivation and self-efficacy, differed based on their background characteristics. Understanding these associations is important, as it helps determine whether health beliefs, which are known to influence preventive behaviors such as cervical cancer screening vary across population subgroups.

This study found that education level was significantly associated with perceived severity of cervical cancer, with women who had primary education reporting higher levels of perceived severity compared to those with secondary or tertiary education. This finding is consistent with the study conducted by Rattay, Michalski (61, 62) which also reported an association between lower educational attainment and higher perceived severity. One possible explanation relates to differences in health literacy, which encompasses the abilities required to access, understand, appraise, and use health-related information and services to make appropriate health decisions (63). Lower levels of education may limit these abilities, particularly in reading, understanding and applying health information effectively (64). Previous studies have consistently identified educational attainment as a strong predictor of health literacy, with individuals of lower educational levels more likely to experience difficulties in accessing, appraising, and interpreting health information. For example, Pasay-an, Saguban (65) found that education influences individuals’ perceptions of illness, their ability to understand health information, and their propensity to engage in self-treatment. This interpretation is further supported by Murfin, Irvine (64) who reported that lower levels of education can constrain health literacy due to limited ability to fully comprehend health-related information. When health literacy is limited, individuals may rely on incomplete or inaccurate information, which may alter their perception of risk and disease severity (66). Similarly, Larki, Tahmasebi (67) highlighted the role of poor health literacy in shaping patients’ understanding of hypertension and its complications, suggesting that limited health literacy may influence how seriously a condition is perceived. Consequently, these findings suggest that improving health literacy through targeted educational strategies may promote a better understanding of cervical cancer among women. This aligns with existing evidence showing that individuals’ levels of cancer literacy influence their engagement in preventive health behaviors, including screening and early detection practices (68–70).

Results from this preliminary study show that women aged above 40 reported significantly higher perceived benefits of cervical cancer screening. This finding is consistent with the study conducted by Alshammari, Alojayri (71) on prostate cancer screening and digital rectal examination. This study reported that older individuals were more likely to participate in prostate cancer screening. This age group also demonstrated a stronger perception of the benefits of screenings and were more health motivated. This may be attributed to greater exposure to health education or increased awareness of age-related health risks. As women grow older, they are more likely to attend routine health check-ups and receive preventive care recommendations, including screening for cervical cancer. Additionally, this age group may have had more opportunities to witness or hear about cervical cancer cases among peers, which could enhance their appreciation of early detection and the protective benefits of screening. Such accumulated experience and exposure likely reinforce the belief that screening is a valuable preventive measure.

Marital status was also found to be significantly associated with perceived susceptibility. Married women tended to perceive themselves at higher risk of developing cervical cancer. Research conducted by Sudenga, Rositch (72) demonstrated that women who perceived themselves to be at risk of cervical cancer were generally older and more likely to be currently or previously married compared to those who did not feel at risk. One possible explanation is their increased likelihood of being sexually active. In many cultures, married women are also more likely to access reproductive health services, where healthcare providers may offer information about cervical cancer and its associated risks. This ongoing interaction with the healthcare system may lead to better understanding of their personal risk and thus, greater perceived susceptibility. Furthermore, social norms or expectations around reproductive health in married life may contribute to higher awareness toward potential health issues like cervical cancer.

Analysis revealed unexpected finding in relation to perceived severity among women who had family members with cervical or other forms of cancer. Rather than reporting higher perceived severity due to proximity to the illness, these women indicated significantly lower levels of perceived severity. A possible explanation is desensitization through exposure, which means when cancer becomes familiar or survivable within a family, it may no longer elicit the same fear or perceived threat. Additionally, seeing relatives cope with or survive the disease might reduce the perception of the disease being severe (73).

### Determinants of screening intention

In relation to screening intention, preliminary findings from this study identified perceived severity and perceived barriers as key determinants of screening intention. Both were found to be negatively associated with the intention to undergo screening. Women with lower screening intention reported significantly higher perceived barriers. These findings are consistent with previous literature, which highlights perceived barriers as a deterrent to cervical cancer screening. Research conducted by Ayu Binti Mohammad Nasir and Lian (7) found that those who perceived fewer barriers to cervical cancer screening were more likely to intend to undergo screening. A study was conducted on the utilization of cervical cancer screening and HBM to determine cervical cancer and screening beliefs among female health care workers (HCW) in Saudi Arabia that involved 1857 participants found that an increase in perceived barriers is associated with lower chances of accepting cervical cancer screening (18).

This study also uncovered that embarrassment about exposing private parts during the Pap smear procedure emerged as the most prominent barrier associated with lower screening intention. This finding reflects prevailing Asian cultural norms that emphasize modesty, privacy and sensitivity surrounding reproductive health, particularly in intimate clinical procedures, which may discourage women from participating in screening (74, 75).

In contrast to perceived barriers, the influence of perceived severity on screening intention presented a more unexpected outcome. This study found that, perceived severity was found to be inversely associated with screening intention (76, 77). Although higher perceived severity is often assumed to act as a motivator for health prevention behaviors like cervical cancer screening, this is not always the case (78). One possible interpretation is that elevated perceptions of severity may be accompanied by emotional responses, such as fear, which could discourage screening. Fear arousal was described as a negative emotional experience that involves physiological, cognitive, emotional, and behavioral reactions in response to perceived threats (79). In this context, perceiving cancer as highly severe may be experienced as threatening, which could lead some individuals to disengage from screening as a way of managing emotional discomfort. Rather than prompting action, heightened perceptions of severity may, in some cases, be associated with defensive responses, particularly when not accompanied by enabling factors. This is reflected in the study’s finding that heightened fear in response to thinking about cervical cancer was associated with lower screening intention. This suggests that, rather than motivating preventive behavior, heightened fear may lead to avoidance or denial, thereby reducing screening uptake, a phenomenon well described in health behavior research where excessive fear can function as a barrier rather than a facilitator to preventive action (80, 81).

These findings highlight the importance of carefully crafted health messages as fear-based messaging could inadvertently induce avoidance rather than motivate action. Recent evidence indicates that presenting information on the severity of possible negative consequence of risky behavior can trigger defensive reactions (79).

## Conclusion

This pilot study explored the relevance of Health Belief Model constructs and evaluated the validity and reliability of the adapted instrument among Malaysian women from lower socioeconomic backgrounds. The findings provide preliminary insights into the relationship between HBM constructs and cervical cancer screening intention, with perceived barriers and perceived severity showing associations with intention within this sample. Although sociodemographic factors were not directly associated with screening intention, their relationships with several HBM constructs suggest a possible indirect influence through cognitive pathways. These findings highlight the importance of addressing perceived barriers and carefully framing perceptions of severity when developing interventions, while also considering variation across population subgroups. Given the exploratory nature of this study and its limited sample size, the findings should be interpreted with caution and are not intended for population-level generalization. Nevertheless, they offer an initial empirical basis for refining the instrument and informing the design of a larger, more representative study, with practical implications for developing targeted strategies to improve screening uptake. In practice, these findings suggest that screening interventions should prioritize reducing practical and emotional barriers that may discourage action. Attention should also be given to how perceptions of severity are communicated, as they may influence women’s willingness to undergo screening. Efforts should therefore focus on supporting women to translate intention into actual screening, particularly among those from lower socioeconomic groups.

### Strength and limitations

While this pilot study offers meaningful insights into the health beliefs influencing cervical cancer screening intention among the lower socioeconomic group women in Malaysia, the limitations should be acknowledged. As a pilot study, aimed to examine and explore the preliminary relevance of Health Belief Model and instrument validity, the sample size was small and participants were recruited using convenience sampling. Importantly, as recruitment was conducted within the Klang Valley, the sample may be more reflective of the urban B40 population from which it was drawn for the pilot study and may not fully represent the broader B40 population across different settings. Therefore, the findings may not be fully representative of all B40 women in Malaysia and may be subject to selection bias. These factors limit the generalizability of the results and the findings observed should be interpreted as preliminary rather than definitive. Nevertheless, the aim of this pilot was to gain preliminary insights into predictors of screening intention rather than to achieve generalizability. Despite this limitation, the study has its strength. It provides an early application of the Health Belief Model in assessing cervical cancer screening intention among Malaysian women, offering practical insights into how theoretical constructs translate in a local context. This contributes valuable groundwork for refining the instrument and informing the design of a larger, adequately powered study.

### Implications

This study presents several implications across theoretical and practical domains. Theoretically, the results offer initial insights into how the Health Belief Model can be used to understand women’s intentions to undergo cervical cancer screening, especially among those from lower socioeconomic backgrounds. The observed associations of perceived barriers and perceived severity with screening intention reinforce the relevance of these constructs, while also suggesting that sociodemographic factors may influence intention indirectly. Practically, the findings highlight the need for interventions that prioritize reducing perceived barriers and carefully framing messages on disease severity to encourage engagement rather than avoidance. Tailored communication strategies may be particularly important in addressing variations across subgroups within the B40 population.

For future research and policy, larger and more representative studies are needed to validate these findings and further examine potential mediating pathways. From a policy perspective, the findings provide useful direction for strengthening cervical cancer screening initiatives under Ministry of Health Malaysia, particularly in refining strategies to address barriers and improve how messages are delivered. Incorporating these considerations into existing programs may help enhance the effectiveness of current efforts to increase screening uptake among lower socioeconomic groups.

## Data Availability

The data for this study is available upon request through the National Institutes of Health – Data Repository System (NIH-DaRS) at https://nihdars.nih.gov.my. Alternatively, requests can be directed to the Sector for Biostatistics & Data Repository, National Institutes of Health Malaysia, via email at nihdars@moh.gov.my, citing the research ID NMRR ID-24-03835-XDC [The dataset for this study is stored in the National Institutes of Health – Data Repository System (NIH-DaRS), Ministry of Health Malaysia, which is a recognized public repository for health research data in Malaysia. In line with Ministry of Health Malaysia requirements, all research conducted under MOH jurisdiction must be submitted to and stored within NIH-DaRS. Although the repository is public, the dataset is treated as restricted health research data under MOH policy. Therefore, it is not openly downloadable. Any request for access must be made through NIH-DaRS and is subject to formal approval by the Ministry of Health Malaysia to ensure compliance with ethical and legal standards, particularly with regard to participant confidentiality and national data governance regulations].
