# Supplementary material for: Health belief model and cervical cancer screening intention among lower socioeconomic women in Malaysia: a pilot study
Source: Front Public Health. 2026 Jun 12;14:1843558. doi: 10.3389/fpubh.2026.1843558 (PMC13303602; doi:10.3389/fpubh.2026.1843558)
Supplement: Supplementary file 1 [file Table_1.DOCX]

**Supplement 1: Research Phases**

PHASE 1A

| **PILOT STUDY** |
| --- |
| To assess the preliminary predictive relevance of Health Belief Model constructs and evaluated the validity and reliability of the adapted instrument. |

PHASE 1B

| **BASELINE: QUANTITATIVE FORMATIVE RESEARCH** |
| --- |
| To determine sociodemographic and HBM factors influencing women’s intention to undergo cervical cancer screening. |

PHASE 2

| **TRIANGULATION: QUALITATIVE RESEARCH** |
| --- |
| To deepen understanding by exploring women’s perceptions related to susceptibility, severity, benefits, barrier, and self-efficacy, and how these shape their motivation to engage in cervical cancer screening. |

PHASE 3

| **DEVELOPMENT OF INTERVENTION** |
| --- |
| To develop an HBM and Behavioural Insights integrated Behaviour Change Communication intervention model suitable to promote and encourage cervical cancer screening uptake among the target population. |

| **IMPLEMENTATION OF INTERVENTION** |
| --- |
| Intervention will be distributed to all the respondents from the baseline survey. |

PHASE 4

| **EVALUATION OF INTERVENTION** |
| --- |
| To evaluate the effectiveness of HBMBI integrated Behaviour Change Communication intervention in improving cervical cancer screening intention and behaviour among the target population |
