# Supplementary material for: Health belief model and cervical cancer screening intention among lower socioeconomic women in Malaysia: a pilot study
Source: Front Public Health. 2026 Jun 12;14:1843558. doi: 10.3389/fpubh.2026.1843558 (PMC13303602; doi:10.3389/fpubh.2026.1843558)
Supplement: Supplementary file 2 [file Table_2.DOCX]

**Supplementary material 2**

**Table 1. Content validity index: the relevance ratings on the item scale by subject matter experts**

| **Item** | **E1** | **E2** | **E3** | **E4** | **E5** | **E6** | **I-CVI^b^** |
| --- | --- | --- | --- | --- | --- | --- | --- |
| Q1 | 1 | 1 | 1 | 1 | 1 | 1 | 1 |
| Q2 | 1 | 1 | 1 | 1 | 1 | 1 | 1 |
| Q3 | 0 | 1 | 1 | 1 | 1 | 1 | 0.83 |
| Q4 | 1 | 1 | 1 | 1 | 1 | 1 | 1 |
| Q5 | 1 | 0 | 1 | 1 | 1 | 1 | 0.83 |
| Q6 | 1 | 0 | 1 | 0 | 1 | 1 | 0.67 |
| Q7 | 1 | 1 | 1 | 1 | 1 | 1 | 1 |
| Q8 | 1 | 1 | 1 | 1 | 1 | 1 | 1 |
| Q9 | 1 | 1 | 1 | 1 | 1 | 1 | 1 |
| Q10 | 1 | 1 | 1 | 1 | 1 | 1 | 1 |
| Q11 | 1 | 1 | 1 | 1 | 1 | 1 | 1 |
| Q12 | 1 | 1 | 1 | 1 | 1 | 1 | 1 |
| Q13 | 1 | 1 | 0 | 1 | 1 | 1 | 0.83 |
| Q14 | 1 | 1 | 1 | 1 | 1 | 1 | 1 |
| Q15 | 1 | 1 | 1 | 1 | 1 | 1 | 1 |
| Q16 | 1 | 1 | 1 | 1 | 1 | 1 | 1 |
| Q17 | 1 | 1 | 1 | 1 | 1 | 1 | 1 |
| Q18 | 1 | 1 | 1 | 1 | 1 | 1 | 1 |
| Q19 | 1 | 1 | 1 | 1 | 1 | 1 | 1 |
| Q20 | 1 | 1 | 1 | 1 | 1 | 1 | 1 |
| Q21 | 1 | 1 | 1 | 1 | 1 | 1 | 1 |
| Q22 | 1 | 1 | 1 | 1 | 1 | 1 | 1 |
| Q23 | 1 | 1 | 1 | 1 | 1 | 1 | 1 |
| Q24 | 1 | 1 | 1 | 1 | 1 | 1 | 1 |
| Q25 | 1 | 1 | 1 | 1 | 1 | 1 | 1 |
| Q26 | 1 | 1 | 1 | 1 | 1 | 1 | 1 |
| Q27 | 1 | 1 | 1 | 1 | 1 | 1 | 1 |
| Q28 | 1 | 1 | 1 | 1 | 1 | 1 | 1 |
| Q29 | 0 | 1 | 1 | 1 | 1 | 1 | 0.83 |
| Q30 | 1 | 1 | 1 | 1 | 1 | 1 | 1 |
| Q31 | 1 | 1 | 1 | 1 | 1 | 1 | 1 |
| Q32 | 0 | 1 | 1 | 1 | 1 | 1 | 0.83 |
| Q33 | 0 | 1 | 1 | 1 | 1 | 1 | 0.83 |
| Q34 | 1 | 1 | 1 | 1 | 1 | 1 | 1 |
| Q35 | 1 | 1 | 1 | 1 | 1 | 1 | 1 |
| Q36 | 1 | 1 | 1 | 1 | 1 | 1 | 1 |
| Q37 | 1 | 1 | 1 | 1 | 1 | 1 | 1 |
| Q38 | 1 | 1 | 1 | 1 | 1 | 1 | 1 |
| Q39 | 1 | 1 | 1 | 1 | 1 | 1 | 1 |
| Q40 | 1 | 1 | 1 | 1 | 1 | 1 | 1 |
| Q41 | 1 | 1 | 1 | 1 | 1 | 1 | 1 |
| Q42 | 1 | 1 | 1 | 1 | 1 | 1 | 1 |
| Q43 | 1 | 1 | 1 | 1 | 1 | 1 | 1 |
| Q44 | 1 | 1 | 1 | 1 | 1 | 1 | 1 |
| Q45 | 1 | 1 | 1 | 1 | 1 | 1 | 1 |
| Q46 | 1 | 1 | 1 | 1 | 1 | 1 | 1 |
| Q47 | 1 | 1 | 1 | 1 | 1 | 1 | 1 |
| Q48 | 1 | 1 | 1 | 1 | 1 | 1 | 1 |
| Q49 | 1 | 1 | 1 | 1 | 1 | 1 | 1 |
| Q50 | 1 | 1 | 1 | 1 | 1 | 1 | 1 |
| Q51 | 1 | 1 | 1 | 1 | 1 | 1 | 1 |
| Q52 | 1 | 1 | 1 | 1 | 1 | 1 | 1 |
|  |  |  |  |  |  |  |  |
| **Content validity index average** | | | | | |  | **0.97** |

Note: **a**=expert; **b**=item-level content validity index

**Table 1. Content validity index: the clarity ratings on the item scale by subject matter experts**

| **Item** | **E1** | **E2** | **E3** | **E4** | **E5** | **E6** | **I-CVI^b^** |
| --- | --- | --- | --- | --- | --- | --- | --- |
| Q1 | 1 | 1 | 1 | 1 | 1 | 1 | 1 |
| Q2 | 1 | 0 | 1 | 1 | 0 | 1 | 0.67 |
| Q3 | 1 | 1 | 1 | 1 | 1 | 1 | 1 |
| Q4 | 1 | 1 | 1 | 0 | 1 | 1 | 0.83 |
| Q5 | 1 | 0 | 1 | 1 | 1 | 1 | 0.83 |
| Q6 | 1 | 0 | 1 | 1 | 1 | 1 | 0.83 |
| Q7 | 1 | 1 | 1 | 1 | 1 | 1 | 1 |
| Q8 | 1 | 1 | 1 | 1 | 1 | 1 | 1 |
| Q9 | 1 | 1 | 1 | 1 | 1 | 1 | 1 |
| Q10 | 1 | 1 | 1 | 1 | 1 | 1 | 1 |
| Q11 | 1 | 1 | 1 | 1 | 1 | 1 | 1 |
| Q12 | 1 | 1 | 1 | 1 | 1 | 1 | 1 |
| Q13 | 1 | 1 | 1 | 1 | 1 | 1 | 1 |
| Q14 | 1 | 1 | 1 | 1 | 1 | 1 | 1 |
| Q15 | 1 | 1 | 1 | 1 | 1 | 1 | 1 |
| Q16 | 1 | 1 | 1 | 1 | 1 | 1 | 1 |
| Q17 | 1 | 1 | 1 | 1 | 1 | 1 | 1 |
| Q18 | 1 | 1 | 1 | 1 | 1 | 1 | 1 |
| Q19 | 1 | 1 | 1 | 1 | 1 | 1 | 1 |
| Q20 | 1 | 1 | 1 | 1 | 1 | 1 | 1 |
| Q21 | 1 | 1 | 1 | 1 | 1 | 1 | 1 |
| Q22 | 1 | 1 | 1 | 1 | 1 | 1 | 1 |
| Q23 | 1 | 1 | 1 | 1 | 1 | 1 | 1 |
| Q24 | 1 | 1 | 1 | 1 | 1 | 1 | 1 |
| Q25 | 1 | 1 | 1 | 1 | 1 | 1 | 1 |
| Q26 | 1 | 1 | 1 | 1 | 1 | 1 | 1 |
| Q27 | 1 | 1 | 1 | 1 | 1 | 1 | 1 |
| Q28 | 1 | 1 | 0 | 1 | 1 | 1 | 0.83 |
| Q29 | 1 | 1 | 1 | 1 | 1 | 1 | 1 |
| Q30 | 1 | 1 | 1 | 1 | 1 | 1 | 1 |
| Q31 | 1 | 1 | 1 | 1 | 1 | 1 | 1 |
| Q32 | 1 | 1 | 1 | 1 | 1 | 1 | 1 |
| Q33 | 1 | 1 | 1 | 1 | 1 | 1 | 1 |
| Q34 | 1 | 1 | 1 | 1 | 1 | 1 | 1 |
| Q35 | 1 | 1 | 1 | 1 | 1 | 1 | 1 |
| Q36 | 1 | 1 | 1 | 1 | 1 | 1 | 1 |
| Q37 | 1 | 1 | 1 | 1 | 1 | 1 | 1 |
| Q38 | 1 | 1 | 0 | 1 | 1 | 1 | 0.83 |
| Q39 | 1 | 1 | 1 | 1 | 1 | 1 | 1 |
| Q40 | 1 | 1 | 1 | 1 | 1 | 1 | 1 |
| Q41 | 1 | 1 | 1 | 1 | 1 | 1 | 1 |
| Q42 | 1 | 1 | 1 | 1 | 1 | 1 | 1 |
| Q43 | 1 | 1 | 1 | 1 | 1 | 1 | 1 |
| Q44 | 1 | 1 | 1 | 1 | 1 | 1 | 1 |
| Q45 | 1 | 1 | 1 | 1 | 1 | 1 | 1 |
| Q46 | 1 | 1 | 1 | 1 | 1 | 1 | 1 |
| Q47 | 1 | 1 | 1 | 1 | 1 | 1 | 1 |
| Q48 | 0 | 1 | 1 | 1 | 1 | 1 | 0.83 |
| Q49 | 1 | 1 | 1 | 1 | 1 | 1 | 1 |
| Q50 | 1 | 1 | 1 | 1 | 1 | 1 | 1 |
| Q51 | 1 | 1 | 1 | 1 | 1 | 1 | 1 |
| Q52 | 1 | 1 | 1 | 1 | 1 | 1 | 1 |
|  |  |  |  |  |  |  |  |
| **Content validity index average** | | | | | | | 0.97 |

Note: **a**=expert; **b**=item-level content validity index

**Table 2. Face validity index: the clarity and comprehension ratings on the item scale by 10 users**

| **Item** | **R1** | **R2** | **R3** | **R4** | **R5** | **R6** | **R7** | **R8** | **R9** | **R10** | **I-FVIb** |
| --- | --- | --- | --- | --- | --- | --- | --- | --- | --- | --- | --- |
| Q1 | 1 | 1 | 1 | 1 | 1 | 1 | 1 | 1 | 1 | 1 | 1 |
| Q2 | 1 | 1 | 1 | 1 | 1 | 1 | 1 | 1 | 1 | 1 | 1 |
| Q3 | 1 | 1 | 1 | 1 | 1 | 1 | 1 | 1 | 1 | 1 | 1 |
| Q4 | 1 | 1 | 0 | 1 | 1 | 1 | 0 | 1 | 1 | 1 | 0.80 |
| Q5 | 1 | 1 | 1 | 1 | 1 | 1 | 1 | 1 | 1 | 1 | 1 |
| Q6 | 1 | 0 | 1 | 0 | 0 | 1 | 1 | 1 | 1 | 1 | 0.70 |
| Q7 | 1 | 1 | 1 | 1 | 1 | 1 | 1 | 1 | 1 | 1 | 1 |
| Q8 | 1 | 1 | 1 | 1 | 1 | 1 | 1 | 1 | 1 | 1 | 1 |
| Q9 | 1 | 1 | 1 | 1 | 1 | 1 | 1 | 1 | 1 | 1 | 1 |
| Q10 | 1 | 1 | 1 | 1 | 1 | 1 | 1 | 1 | 1 | 1 | 1 |
| Q11 | 1 | 1 | 1 | 1 | 1 | 1 | 1 | 1 | 1 | 1 | 1 |
| Q12 | 1 | 1 | 1 | 1 | 1 | 1 | 1 | 1 | 1 | 1 | 1 |
| Q13 | 1 | 1 | 1 | 1 | 1 | 1 | 1 | 1 | 1 | 1 | 1 |
| Q14 | 1 | 0 | 0 | 1 | 1 | 1 | 0 | 1 | 1 | 1 | 0.70 |
| Q15 | 1 | 0 | 0 | 1 | 1 | 1 | 0 | 1 | 1 | 1 | 0.70 |
| Q16 | 1 | 1 | 1 | 1 | 1 | 1 | 1 | 1 | 1 | 1 | 1 |
| Q17 | 1 | 1 | 1 | 1 | 1 | 1 | 1 | 1 | 1 | 1 | 1 |
| Q18 | 1 | 1 | 1 | 1 | 1 | 1 | 1 | 1 | 1 | 1 | 1 |
| Q19 | 1 | 0 | 0 | 1 | 1 | 1 | 1 | 1 | 1 | 1 | 0.80 |
| Q20 | 1 | 1 | 1 | 1 | 1 | 1 | 1 | 1 | 1 | 1 | 1 |
| Q21 | 1 | 1 | 1 | 1 | 1 | 1 | 1 | 1 | 1 | 1 | 1 |
| Q22 | 1 | 0 | 0 | 1 | 1 | 1 | 1 | 1 | 1 | 1 | 0.80 |
| Q23 | 1 | 1 | 1 | 1 | 1 | 1 | 1 | 1 | 1 | 1 | 1 |
| Q24 | 1 | 1 | 1 | 1 | 1 | 1 | 1 | 1 | 1 | 1 | 1 |
| Q25 | 1 | 1 | 0 | 1 | 1 | 1 | 1 | 1 | 1 | 1 | 0.90 |
| Q26 | 1 | 1 | 0 | 0 | 0 | 1 | 1 | 1 | 1 | 1 | 0.70 |
| Q27 | 1 | 1 | 1 | 1 | 1 | 1 | 1 | 1 | 1 | 1 | 1 |
| Q28 | 1 | 1 | 1 | 1 | 1 | 1 | 1 | 1 | 1 | 1 | 1 |
| Q29 | 1 | 1 | 1 | 1 | 1 | 1 | 1 | 1 | 1 | 1 | 1 |
| Q30 | 1 | 1 | 1 | 1 | 1 | 1 | 1 | 1 | 1 | 1 | 1 |
| Q31 | 1 | 1 | 1 | 1 | 1 | 1 | 1 | 1 | 1 | 1 | 1 |
| Q32 | 1 | 1 | 1 | 1 | 1 | 1 | 1 | 1 | 1 | 1 | 1 |
| Q33 | 1 | 1 | 1 | 1 | 1 | 1 | 1 | 1 | 1 | 1 | 1 |
| Q34 | 1 | 1 | 1 | 1 | 1 | 1 | 1 | 1 | 1 | 1 | 1 |
| Q35 | 1 | 1 | 1 | 1 | 1 | 1 | 1 | 1 | 1 | 1 | 1 |
| Q36 | 1 | 1 | 1 | 1 | 1 | 1 | 1 | 1 | 1 | 1 | 1 |
| Q37 | 1 | 1 | 1 | 1 | 1 | 1 | 1 | 1 | 1 | 1 | 1 |
| Q38 | 1 | 1 | 1 | 1 | 1 | 1 | 1 | 1 | 1 | 1 | 1 |
| Q39 | 1 | 1 | 1 | 1 | 1 | 1 | 1 | 1 | 1 | 1 | 1 |
| Q40 | 1 | 1 | 1 | 1 | 1 | 1 | 1 | 1 | 1 | 1 | 1 |
| Q41 | 1 | 1 | 1 | 1 | 1 | 1 | 1 | 1 | 1 | 1 | 1 |
| Q42 | 1 | 1 | 1 | 1 | 1 | 1 | 1 | 1 | 1 | 1 | 1 |
| Q43 | 1 | 1 | 1 | 1 | 1 | 1 | 1 | 1 | 1 | 1 | 1 |
| Q44 | 1 | 1 | 1 | 1 | 1 | 1 | 1 | 1 | 1 | 1 | 1 |
| Q45 | 1 | 1 | 1 | 1 | 1 | 1 | 1 | 1 | 1 | 1 | 1 |
| Q46 | 1 | 1 | 1 | 1 | 1 | 1 | 1 | 1 | 1 | 1 | 1 |
| Q47 | 1 | 1 | 1 | 1 | 1 | 1 | 1 | 1 | 1 | 1 | 1 |
| Q48 | 1 | 1 | 1 | 1 | 1 | 1 | 1 | 1 | 1 | 1 | 1 |
| Q49 | 1 | 1 | 1 | 1 | 1 | 1 | 1 | 1 | 1 | 1 | 1 |
| Q50 | 1 | 1 | 1 | 1 | 1 | 1 | 1 | 1 | 1 | 1 | 1 |
| Q51 | 1 | 1 | 1 | 1 | 1 | 1 | 1 | 1 | 1 | 1 | 1 |
| Q52 | 1 | 1 | 1 | 1 | 1 | 1 | 1 | 1 | 1 | 1 | 1 |
|  |  |  |  |  |  |  |  |  |  |  |  |
| **Face validity index average** | | | | | | | | | | | 0.96 |

Note: **a**=respondent; **b**=item-level face validity index
